# NF-κB signaling regulates myelination in the CNS

**DOI:** 10.3389/fnmol.2014.00047

**Published:** 2014-05-26

**Authors:** Thomas Blank, Marco Prinz

**Affiliations:** ^1^Institute of Neuropathology, University of FreiburgFreiburg, Germany; ^2^BIOSS Centre for Biological Signalling Studies, University of FreiburgFreiburg, Germany

**Keywords:** NF-κB pathway, myelin, oligodendrocyte, demyelination, remyelination, oligodendrocyte precursor cells

## Abstract

Besides myelination of neuronal axons by oligodendrocytes to facilitate propagation of action potentials, oligodendrocytes also support axon survival and function. A key transcription factor involved in these processes is nuclear factor-κB (NF-κB), a hetero or homodimer of the Rel family of proteins, including p65, c-Rel, RelB, p50, and p52. Under unstimulated, NF-κB remains inactive in the cytoplasm through interaction with NF-κB inhibitors (IκBs). Upon activation of NF-κB the cytoplasmic IκBs gets degradated, allowing the translocation of NF-κB into the nucleus where the dimer binds to the κB consensus DNA sequence and regulates gene transcription. In this review we describe how oligodendrocytes are, directly or indirectly via neighboring cells, regulated by NF-κB signaling with consequences for innate and adaptive immunity and for regulation of cell apoptosis and survival.

## INSULATING AXONS VIA OLIGODENDROCYTES

**FIGURE 1:**
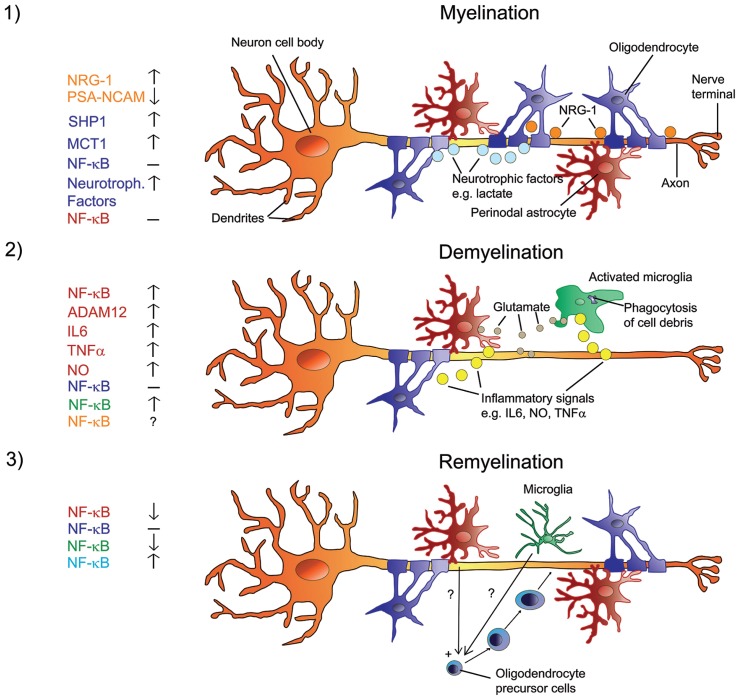
**Signals in the regulation of myelination, de- and remyelination in the CNS with a focus on NF-κB.** Myelination involves a sequence of orchestrated steps, where PSA-NCAM is downregulated in neurons and astrocytes, and neurons release several growth/trophic factors such as NRG-1 and regulate oligodendrocyte survival and maturation by upregulating SHP1. Oligodendrocytes provide trophic support to axons and promote their viability via upregulation of MCT1 and the release of lactate. There is no activation of NF-κB in astrocytes or oligodendrocytes **(1)**. When myelinated axons undergo demyelination, myelin debris is phagocytized by microglia. Resident astrocytes and microglia get activated and produce glutamate in addition to inflammatory signals such as IL6, NO, ADAM12, and TNFα. Activated astrocytes and microglia show elevated NF-κB activation and produce factors that activate each other. The role of NF-κB in neurons is unclear at present. NF-κB in oligodendrocytes is not activated **(2)**. Under the influence of yet unknown factors that are produced by non-activated microglia and potentially by non-activated astrocytes, NF-κB is activated in recruited oligodendrocyte progenitor cells that engage demyelinated axons and differentiate into remyelinating oligodendrocytes **(3)**. Color code on the left represents cell type.

Oligodendrocytes are specialized cells in the CNS that wrap multiple axons with myelin, forming as many as 40 separate myelin segments (Salzer, 2003). This specialization of glial cells was the last major phylogenetical invention for the nervous system of vertebrates (Zalc et al., 2008). A series of stages that result from reciprocal, axo-glial interactions are necessary for the development of myelinated fibers. For the initial step of myelin formation, immature post-mitotic oligodendrocytes need to extend numerous cytoplasmic protrusions (filipodia) in order to find suitable myelin-competent axons. As microfilament-rich filipodia extend they are invaded by microtubules, thus further enlarging these processes and converting them to lamellipodia. The oligodendrocyte cytoskeleton now increases microfilament polymerization and branching in response to axonal signals. The majority of axonal signals identified to date is expressed to prevent the initiation of myelination and/or exuberant over-myelination. One example includes axonal PSA-NCAM, which is developmentally downregulated to coincide with the onset of myelination (Decker et al., 2000; Fewou et al., 2007). To ensure that glial numbers are matched to axon length, axon outgrowth regulates gliogenesis via mitogenic and trophic effects (Barres and Raff, 1999). But interactions between axonal ligands and glial receptors have to be integrated to modulate myelination and myelin thickness. But what are the exact signals? The protein tyrosine phosphatase Src homology region 2 domain-containing phosphatase-1 (SHP-1) is a critical regulator of developmental signals leading to terminal differentiation and myelin sheath formation by oligodendrocytes; Figure 1). The SHP-1 homolog, SHP-2, regulates oligodendrocyte progenitor proliferation (Kuo et al., 2010). ErbB, the neuronal growth factor receptor of Neuregulin-1 (NRG-1), affects oligodendrocyte specification after binding to its receptor (Figure 1). It further regulates differentiation, myelination, and survival, at least *in vitro* (Canoll et al., 1996; Vartanian et al., 1997; Calaora et al., 2001). The situation *in vivo* is more complex. Only minor effects on overall myelination in the CNS of mice were reported for knockout of NRG1, whereas a significant hypermyelination was achieved by transgenic overexpression of NRG1 (Brinkmann et al., 2008). However, in a more recent study, hypomyelination and thinner myelin sheaths were found in the prefrontal cortex when NRG-1 signaling was disturbed (Makinodan et al., 2012). It should be mentioned that neuronal activity can regulate NRG-1 levels thus linking it to myelin production (Ziskin et al., 2007; Liu et al., 2011). Myelin sheaths are radially organized with distinct proteins in the abaxonal (e.g., oligodendrocyte myelin glycoprotein) and inner glial (e.g., MAG and NCAM) membranes.

Myelination of axons allows for the process of saltatory conduction, in which a neuronal action potential is propagated between nodes of Ranvier to increase both the speed and energy efficiency of nerve conduction. The generation of action potentials (AP) is possible due to the accumulation of voltage gated sodium channels, Na^+^/K^+^ ATPases, Na^+^/Ca^2^^+^ antiporters, as well as specific subtypes of potassium channels important for the regulation of repetitive discharges (Devaux et al., 2004; Pan et al., 2006). Myelination also markedly decreases the refractory time (time needed for repolarization before a new AP can be supported by the axon; Felts et al., 1997; Sinha et al., 2006). Qualitative differences of myelination along axons, such as variations in internode distance and myelin sheath thickness, enable systematic regulation of conduction velocity. Myelination sometimes ensures that axons of different length have isochronous conduction times, enabling them to activate their synaptic targets simultaneously. Two examples include the projections of retinal ganglion neurons to the lateral geniculate (Stanford, 1987) and projections of inferior olivary neurons to Purkinje cells in the cerebellar cortex (Sugihara et al., 1993). Thus, intact myelin enhances the integration of information across spatially distributed neural networks supporting cognitive and motor functions (Bartzokis et al., 2001; Lutz et al., 2005). There is further increasing evidence that oligodendrocytes provide trophic support to axons and promote their viability. These mechanisms may include metabolic coupling, with oligodendrocytes providing axons with lactate as an energy source via the lactate transporter monocarboxylate transporter 1 (MCT1, also known as SLC16A1; Funfschilling et al., 2012; Lee et al., 2012b).

Nuclear factor-κB is a ubiquitously expressed dimeric molecule that regulates the expression of a variety of genes and has a key role in a number of cellular processes such as innate and adaptive immunity, cellular proliferation, apoptosis, and development. Often diseases involving oligodendrocytes are associated with NF-κB activation causing some degree of demyelination. Whether this effect is of direct nature or indirect via surrounding cells and the potential contribution of NF-κB to phenomena like myelination and remyelination will be carefully highlighted and discussed in the following paragraphs.

## IS NF-κB ESSENTIAL FOR MYELINATION IN THE CNS?

Originally identified as a transcription factor that regulates expression of the immunoglobulin kappa light chain gene in response to cytokine stimulation in B lymphocytes, NF-κB is now known to be expressed in most, if not all, mammalian cells. Five subunits, p50, p52, p65 (RelA), RelB, and c-Rel, form homo and heterodimers (Karin and Lin, 2002; Yan and Greer, 2008). In an inactive state, NF-κB dimers are sequestered in the cytoplasm by the specific inhibitors IκBα, IκBβ, and IκBε. On stimulation, IκB is phosphorylated by the IκB kinase (IKK) complex, ubiquitinated, and then degraded by the 26S proteasome. The IKK complex contains two enzymatic subunits, IKK1 (also known as IKKα) and IKK2 (also known as IKKβ), with partially overlapping substrate specificity. IKK2 is required for NF-κB activation through the canonical pathway triggered by proinflammatory stimuli. These diverse stimuli that activate NF-κB do not only include inflammatory cytokines such as tumor necrosis factor-α (TNF-α) but also neurotrophic factors such as nerve growth factor (NGF), neurotransmitters, cell adhesion molecules and various types of cell stress (Mattson and Camandola, 2001; Karin and Lin, 2002). Genes that can be induced by NF-κB include those that encode cytokines such as TNF-α and interleukin-1β, interleukin 6, the antioxidant enzyme manganese superoxide dismutase, and the anti-apoptotic protein Bcl-2 (Mattson and Camandola, 2001). At present, there is ongoing controversial discussion about the contribution of NF-κB to myelin production in the CNS. In a recent study it was shown that in patients with additional copies of the IKBKG gene, which encodes for NF-κB essential modulator (NEMO), the regulatory subunit of the IKK complex, NF-κB signaling is impaired. These patients showed defective myelination, developmental brain abnormalities and mild mental retardation (Philippe et al., 2013). From these findings it was concluded that proper myelination in the CNS requires NF-κB activation. However, several transgenic mouse studies seem to show exact the opposite. When the NF-κB subunit RelA was almost completely deleted from the mouse CNS, histological and electron microscopic analyses showed unimpaired oligodendrocyte densities and normal myelin sheath formation (Kretz et al., 2014). Accordingly, mice with inactivated NF-κB by either overexpression of the super repressor IκB*α* or deletion of the activator IKKβ in the neuro-glial compartment develop normally and display no alterations in overall neuro-anatomical and behavioral features (Herrmann et al., 2005; Zhang et al., 2005). There is further evidence that IKKβ-mediated NF-κB activation is dispensable for oligodendrocyte maturation *in vitro* and *in vivo*, and subsequent insulation of axons in the CNS (Raasch et al., 2011). These results are either in sharp contrast to the reported crucial role of NF-κB signaling for myelination in the PNS (Nickols et al., 2003), or display a similar picture as seen in mice with ablated IKKβ in Schwann cells, where NF-κB activation was described as dispensable for myelination (Morton et al., 2013). Since the study by Nickols et al. (2003) was solely performed using cultured neurons, a possible explanation for the discrepancy between these findings might be that Schwann cells simply behave differently *in vivo* and *in vitro*. Thus, NF-κB signaling appears expendable for myelination in the PNS and CNS.

## NF-κB, DE-AND REMYELINATION IN THE CNS

Among the NF-κB family members only deletion of p50 results in spontaneous demyelination in young adult animals (Lu et al., 2006). Electron microscopy revealed an age-dependent reduction in the number of axons and degenerative alterations in the optic nerve of both wild type and p50^-^^/^^-^ mice. P50^-^^/^^-^ knockout markedly accelerated the axonal reduction and degeneration most likely due to demyelination as well as axonal degeneration. This effect on myelination or axonal degeneration cannot be explained by reduced NF-κB activity since p50 deficiency in mice enhances NF-κB activity (Schmitz and Baeuerle, 1991; Tang et al., 2010). Accordingly, in mice with conditional neuronal NF-κB ablation, the clinical course of experimental autoimmune encephalomyelitis (EAE), a well-characterized animal model mimicking multiple sclerosis in humans, parameters of inflammation and axonal densities in the spinal cord white and gray matter were not different to littermate controls (Lee et al., 2012a). In a similar approach using genetically engineered mice, a cell type specific knockout of NF-κB essential modulator (NEMO) or IκBα-kinase-complex (IKK)-2 with Nestin promoter-driven Cre expression ameliorated EAE (van Loo et al., 2006). Here the NF-κB regulated expression of pro-inflammatory cytokines and cell adhesion molecules in astrocytes rather than effects in neurons were found essential for the propagation of EAE. In the only real contrasting study a neuroprotective effect of neuronal IKK-2 in autoimmune demyelination was reported (Emmanouil et al., 2011). It cannot be excluded that the observed effects are due to potentially other, so far unknown, phosphorylation targets of IKK-2 than IκB. *In vitro* studies have shown that NF-κB exhibits a pro-survival role in a rat oligodendrocyte precursor cell line, with p50 being more effective than p65 to prevent TNF-α-induced apoptosis (Hamanoue et al., 2004). NF-κB further promotes survival and maturation of oligodendrocyte progenitor cells *in vitro* (Nicholas et al., 2001), a finding that was not apparent by our *in vivo* study where we found a normal number of mature oligodendrocytes in the adult corpus callosum of IKK2-deficient brains (Raasch et al., 2011). Importantly, brain-specific IKK2-dependent NF-κB signaling has an essential role during toxin-induced demyelination *in vivo*. The amelioration of demyelination in mice with brain-restricted NF-κB inhibition correlated with impaired induction of inflammatory cytokines, which are potentially toxic for oligodendrocytes. This protection against demyelination was mediated through ablation of IKK2 from astrocytes but not from oligodendrocytes (Raasch et al., 2011). Astrocytic NF-κB inhibition would also diminish expression of A disintegrin and metalloproteinase (ADAM) 12, which showed elevated expression especially in brain regions affected by oligodendrocyte loss (Baertling et al., 2010). An increased number of ADAM12-positive astrocytes was for example observed after the induction of toxic demyelination by cuprizone feeding. Whether this is ultimately of detrimental or supportive nature for oligodendrocytic function has to be determined. ADAM12 has been shown to cleave insulin-like growth factor-2 binding protein-3 (Shi et al., 2000). This protein is obviously functionally related to the development of oligodendrocytes and the formation and/or regeneration of the myelin sheath (Mewar and McMorris, 1997; Lovett-Racke et al., 1998). At present, however, it remains elusive which functions ADAM12 takes over during de- and remyelination.

Astrocytic, but not microglial NF-κB inhibition was also responsible for protection against cuprizone-induced demyelination by the new oral immunomodulatory compound Laquinimod (LAQ; Bruck et al., 2012). This finding might seem surprising considering that during cuprizone-induced demyelination NF-κB was activated not only in astrocytes but also in microglia (Millet et al., 2009). Indeed, mice with a selective inactivation of the NF-κB pathway in microglia were substantially protected from the induction of EAE (Goldmann et al., 2013). The loss of oligodendrocytes may be replaced by proliferating nerve/glial antigen 2 (NG2) cells, also known as oligodendrocyte precursor cells (Tripathi and McTigue, 2007). These OPCs are able to migrate to the damaged site and differentiate into mature myelinating oligodendrocytes if the environment is permissive (Franklin and Ffrench-Constant, 2008). For instance, degenerated myelin contains inhibitory molecules such as NogoA, Oligodendrocyte-myelin glycoprotein (OMgp) and myelin-associated glycoprotein (MAG). Degenerated myelin further activates the FAK/PI3K/Akt/NF-κB pathway in macrophages and increases the expression of inflammatory mediators (Sun et al., 2010). These factors inhibit axon regeneration and further activate complement systems to destroy intact myelin (McKerracher et al., 1994; Chen et al., 2000). In transgenic mice with NF-κB inhibition specifically in astrocytes an increase in oligodendrogenesis was observed following spinal cord injury (Bracchi-Ricard et al., 2013). The same mice were significantly protected against optic neuritis and showed a nearly complete prevention of axonal demyelination, as well as a drastic attenuation in retinal ganglion cell death (Brambilla et al., 2012). Following EAE induction, NOS2 and the NAD(P)H oxidase subunits Cybb/NOX2 and Ncf1 were upregulated in WT mice but not in GFAP-IκBα-dn mice, where NF-κB is selectively inactivated in astrocytes. On the other hand, activation of the NF-κB pathway in oligodendrocytes contributes to the protective effects of enhanced pancreatic endoplasmic reticulum kinase (PERK) signaling during EAE including reduced oligodendrocyte apoptosis, demyelination, and axonal degeneration (Deng et al., 2004). PERK signaling activates NF-κB, an antiapoptotic transcription factor, by repressing the translation of IκBα, an inhibitor of NF-κB (Lin et al., 2013).

These GFAP-IκBα-dn mice showed not only preservation of myelin compaction but also enhanced remyelination during recovery from EAE due to reduced expression of pro-inflammatory genes (Brambilla et al., 2014). In the same line, activation of NF-κB within astrocytes resulted in a significant increase in oligodendrocyte death following trauma by reducing extracellular zinc levels and inducing glutamate excitotoxicity (Johnstone et al., 2013). These results are consistent with several *in vitro* studies which indicated that astrocytes can directly modulate myelination via the release of a number of secreted factors, depending on culture conditions (Moore et al., 2011; Nash et al., 2011). From the *in vivo* data, however, it could not be determined whether the decreased expression of an NF-κB-regulated gene has a direct effect on oligodendrocyte maturation or an indirect effect through other cells such as microglia and/or infiltrating macrophages. Indeed, it was speculated that inhibiting astroglial NF-κB affects the activation status of microglia/leukocytes rendering them more supportive for remyelination. From *in vitro* experiments it was reported that non-activated microglia activate NF-κB in OPCs thereby increasing the number of surviving oligodendrocytes by inhibiting the apoptosis of OPCs and stimulating their maturation to oligodendrocytes (Nicholas et al., 2001). In accordance, when mice were fed cuprizone together with low concentrations of lactacystin, a specific inhibitor of the 26S proteasome, they showed a decrease in activated microglia response with a markedly diminished NF-κB activation during their remyelination period when compared to mice fed cuprizone only (Millet et al., 2009). Ineffective remyelination is often caused by the presence of differentiation inhibitors in the vicinity of oligodendrocyte damage, which include cytokines and chemokines, many of which are regulated by NF-κB (Kotter et al., 2006; Kremer et al., 2011). Although NF-κB might also support the remyelination process via TNF, a prototypical inducer of NF-κB, which is required for both remyelination and proliferation of OPCs (Arnett et al., 2001; Figure 1), we found no active role for oligodendrocyte-specific IKK2 during remyelination (Raasch et al., 2011). Inhibition of NF-κB in Schwann cells also seems to have no major impact on remyelination except for transiently slowing down the whole process (Morton et al., 2012).

Taken together, the present data suggest no direct contribution of the oligodendrocytic NF-κB pathway to myelination, de- and remyelination. Activation of astrocytic and microglial NF-κB, however, seems to favor demyelination whereas its inhibition supports remyelination. Activation of NF-κB in OPCs increases the number of surviving oligodendrocytes and stimulates their maturation to oligodendrocytes which effects myelination and remyelination.

## Conflict of Interest Statement

The authors declare that the research was conducted in the absence of any commercial or financial relationships that could be construed as a potential conflict of interest.
